# Modified adeno‐associated virus targets the bacterial enzyme chondroitinase ABC to select mouse neuronal populations in vivo using the Cre‐LoxP system

**DOI:** 10.1111/ejn.15050

**Published:** 2020-12-20

**Authors:** Kelly E. Carstens, Bernd R. Gloss, Georgia M. Alexander, Serena M. Dudek

**Affiliations:** ^1^ Neurobiology Laboratory National Institute of Environmental Health Sciences NIH Durham NC USA

**Keywords:** hippocampal area CA2, hippocampus, mossy fibers, mouse, perineuronal nets, plasticity, PNNs

## Abstract

Current methods of experimentally degrading the specialized extracellular matrix (ECM), perineuronal nets (PNNs) have several limitations. Genetic knockout of ECM components typically has only partial effects on PNNs, and knockout of the major ECM component aggrecan is lethal in mice. Direct injection of the chondroitinase ABC (ChABC) enzyme into the mammalian brain is effective at degrading PNNs in vivo but this method typically lacks consistent, localized spatial targeting of PNN degradation. PNNs also regenerate within weeks after a ChABC injection, thus limiting the ability to perform long‐term studies. Previous work has demonstrated that viral delivery of ChABC in mammalian neurons can successfully degrade PNNs for much longer periods, but the effects are similarly diffuse beyond the injection site. In an effort to gain cell‐specific targeting of ChABC, we designed an adeno‐associated virus encoding ChABC under the control of the Cre‐LoxP system. We show that this virus is effective at targeting the synthesis of ChABC to Cre‐expressing mouse neurons in vivo. Although ChABC expression is localized to the Cre‐expressing neurons, we also note that ChABC is apparently trafficked and secreted at projection sites, as was previously reported for the non‐Cre dependent construct. Overall, this method allows for cell‐specific targeting of ChABC and long‐term degradation of PNNs, which will ultimately serve as an effective tool to study the function of cell‐autonomous regulation of PNNs in vivo. This novel approach may also aid in determining whether specific, long‐term PNN loss is an appropriate strategy for treatment of neurodevelopmental disorders associated with PNN pathology.

## Graphical abstract

**Cre‐Dependent AAV‐ChABC Construct**: To express chondroitinaseABC (ChABC) in a Cre‐recombinase dependent way, a shuttle plasmid containing a double floxed mCherry under the control of human synapsin promoter was used for the viral vector backbone, pAAV‐hSyn‐DIO‐mCherry. We tested this virus in mice expressing a tamoxifen‐regulated Cre recombinase in hippocampal area CA2.fx1

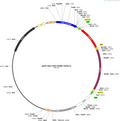

**Abbreviations**
AAV: adeno‐associated virus
CA2: *Cornu Ammonis* 2
ChABC: chondroitinase ABC
IP: intraperitoneal
PNNs: perineuronal nets
WFA: *Wisteria floribunda* agglutinin

## 1 INTRODUCTION

The function of the specialized extracellular matrix (ECM), perineuronal nets (PNNs), has been effectively studied by degrading PNNs in vivo and in vitro using the exogenous enzyme chondroitinase ABC (ChABC). ChABC is a bacterial enzyme that catalyzes the degradation of the N‐glycosaminoglycan chains of chondroitin sulfate proteoglycans (CSPGs). ChABC has been found to improve functional recovery from spinal cord injury by targeting the upregulation of CSPGs and promoting structural plasticity at the site of injury (Bradbury et al., 2002; Galtrey et al., 2007; Rosenzweig et al., 2019). In the brain, ChABC‐mediated digestion of PNNs in vivo is sufficient to re‐open critical windows of plasticity in a number of brain regions (Gogolla et al., 2009; Pizzorusso et al., 2002; Romberg et al., 2013). The mechanism(s) by which PNNs suppress plasticity remains unclear. Many studies suggest that PNNs play a role in modulating inhibitory transmission in regions where they predominantly surround inhibitory neurons (Fawcett et al., 2019). We previously reported a role for PNNs in restricting synaptic plasticity in a population of excitatory neurons in hippocampal area CA2 in vitro (Carstens et al., 2016; Zhao et al., 2007). In order to address questions related to the functional role of PNNs surrounding CA2 neurons, we sought to selectively degrade PNNs in CA2 in vivo.

The current methods of experimentally degrading PNNs in vivo have several limitations. For example, knockout of the major ECM component, aggrecan, is lethal during embryonic development, thus limiting study to those experiments that can be performed in vitro (Giamanco et al., 2010). Moreover, knockout of a single PNN glycoprotein, such as tenascin‐R, is insufficient to fully reduce staining for this matrix, although these mice do exhibit aberrant synaptic and structural plasticity (Brakebusch et al., 2002; Carulli et al., 2010; Geissler et al., 2013; Saghatelyan et al., 2001). Direct injection of ChABC in the mammalian brain is effective, but typically lacks localized and consistent spatial targeting of PNNs. PNN loss often appears widespread from the point of injection and the radius of degradation is variable between studies. Using a viral approach, previous work demonstrated successful synthesis of the ChABC enzyme in mammalian neurons using adeno‐associated virus (AAV) or lentiviral vectors (Alves et al., 2014; Jin et al., 2011; Zhao et al., 2011). This experimental approach effectively degrades PNNs in vivo but is similarly limited in targeting of PNN degradation; the viral uptake lacks cell‐specificity, targeted localization, and the construct lacks a reporter tag to validate the expression of ChABC. In an attempt to overcome these limitations and target PNNs to specific cell populations, we created a tool to express ChABC using the Cre‐LoxP system. Here we describe an inducible approach, using hippocampal CA2 pyramidal neurons as an example that allows for labeled targeting of ChABC with cell‐type specificity in vivo and effective, localized degradation of PNNs.

## 2 MATERIALS AND METHODS

### 2.1 Animals

Male mice, approximately 12 weeks of age, were group‐housed under a 12:12 light/dark cycle with access to food and water *ad libitum*. All procedures were approved by National Institute of Environmental Health Sciences (NIEHS) Animal Care and Use Committee and were in accordance with the United States National Institutes of Health guidelines for care and use of animals.

### 2.2 Generation of a Cre‐dependent AAV‐ChABC‐P2A‐mCherry

**FIGURE 1:**
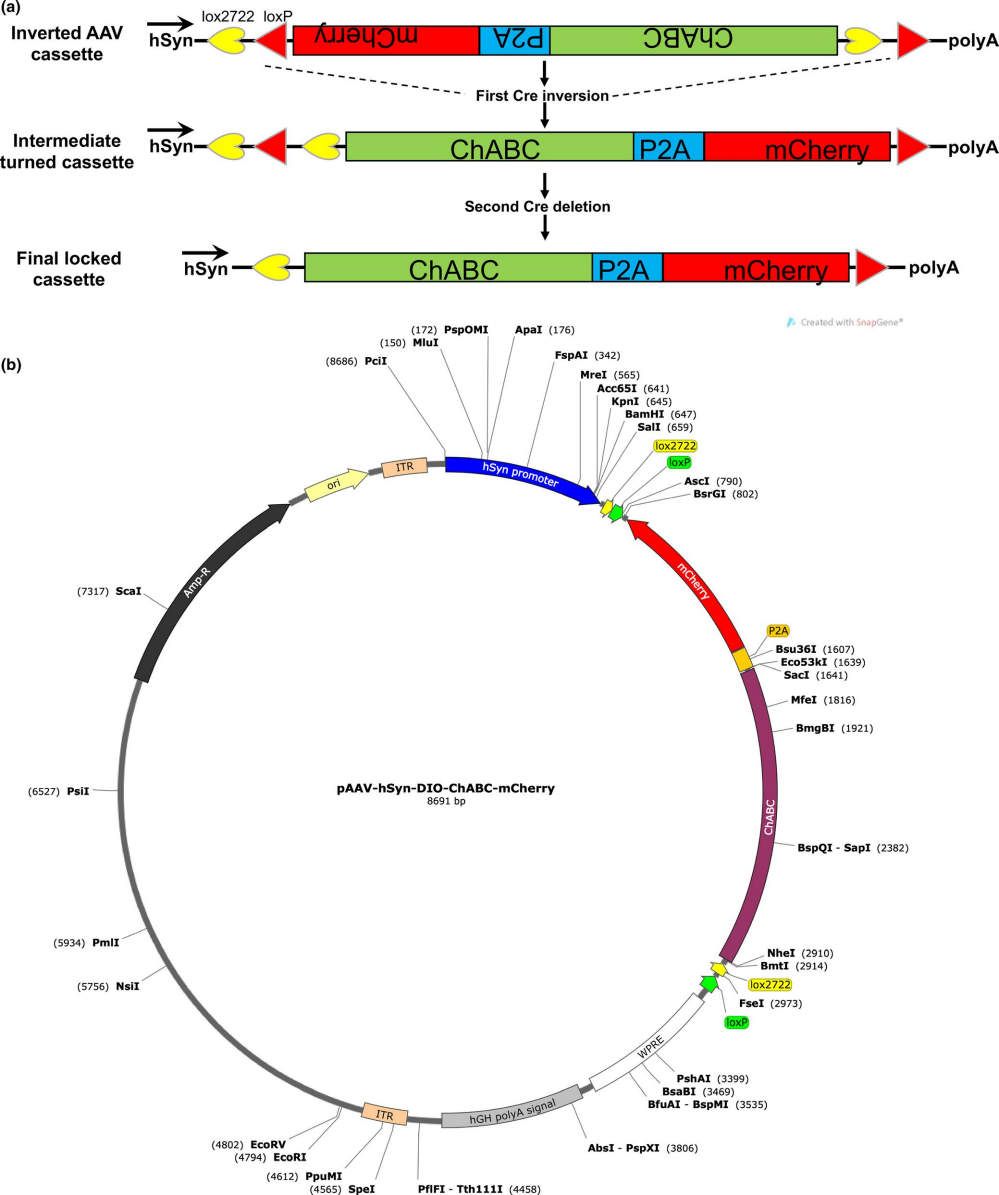
Strategy for Cre‐dependent expression of ChABC by the human synapsin promoter (hSyn). (a) This schematic shows the two‐step inversion of the ChABC open reading frame by the action of Cre recombinase. The Cre enzyme is able to invert any DNA sequence that is flanked by two identical loxP sites in a head to head orientation, while the Cre enzyme deletes any DNA between head to tail loxP sites. LoxP sites are depicted by a red arrow and a yellow heart. In the first step the entire open reading frame of ChABC‐P2A‐mCherry including the two loxP sites is inverted to yield the intermediate turned cassette. The second action of Cre then deletes the sequence between the head to tail oriented lox2722 sites shown as yellow hearts. This leads to the final locked cassette containing two different loxP sites that cannot be recombined anymore by the Cre enzyme. (b) Illustrated is the Cre‐dependent AAV‐ChABC construct. A shuttle plasmid containing a double floxed mCherry under the control of human synapsin promoter was used for viral vector backbone, pAAV‐hSyn‐DIO‐mCherry

In an effort to degrade PNNs with cell‐specificity, we designed a Cre‐dependent AAV that encodes the PNN‐degrading enzyme ChABC with a cleavable mCherry fluorescent protein linked to ChABC. The Cre‐loxP system can be used to specifically express a double‐floxed gene cassette in all cells that contain the Cre enzyme. The double‐floxed inverse orientation (DIO) arrangement takes advantage of the principle that the Cre enzyme is able to invert any DNA sequence that is flanked by two identical loxP sites in a head to head orientation, while the Cre enzyme deletes any DNA between head to tail loxP sites (Schnutgen et al., 2003; Atasoy et al., 2008). Orientation of the palindromic 34 bp loxP site comes about by a unique spacer sequence that separates the 13‐bp palindromes, ATGTATGC. In the variant lox2722 site that is also used here, the sequence reads AAGTATCC. In this AAV virus, the ChABC‐P2A‐mCherry cassette is flanked by both a loxP and a lox2722 site, such that when the cassette is inverted, two loxP sites will be flipped into a head to tail configuration and the intervening sequence between the head to tail loxP sites is deleted leaving only one loxP site behind. This loxP site is paired with the variant lox2722 site at the other end of the cassette, and the Cre enzyme does not invert or recombine the two distinct lox sites, thereby locking the cassette in one orientation that allows expression by the hSyn promoter. We also encoded a cleavable fluorescent reporter protein, mCherry, in the gene cassette via a P2A site such that mCherry will be expressed in cells that synthesize the ChABC enzyme, but not bound to the ChABC protein (Figure 1a).

### 2.3 Chondroitinase gene

For the ChABC gene insert, we used the synthetic chondroitinase gene sequence “Y133”’ that was published in lentiviral‐ChABC studies (Zhao et al., 2011). Originally this sequence was optimized for enzymatic secretion from mammalian cells in vitro and in tissue culture (Muir et al., 2010) and was further modified as described in (Zhao et al., 2011), which includes an MMP3 signal sequence. The ChABC sequence containing a P2A processing sequence at its 3’ end was cloned into vector pUC57 at the Xba I site (GenScript, Piscataway, NJ). The synthetic ChABC cDNA sequence, sequence insert, 3162 base pairs, is as follows, and includes Xba I restriction sties (XbaI; underlined), KOZAK sequence to initiate translation (KOZAK; ***bold italic***) and P2A sequences to cleave ChABC from mCherry (**P2A; in bold caps**), used for cloning into pAAV‐hSyn‐DIO‐mCherry. The chondroitinase (ChABC) sequence is unformatted.

5′‐tctaga***gccgccacc*** atggaagcccgtgtagcgtggggagccctagcaggccctttgcgggtcctttgcgttctgtgctgtctacttggtcgtgccatagcggcaactagtaatcccgccttcgaccccaaaaacctgatgcagagcgaaatctaccatttcgcccagaataatcctctcgcagacttcagcagtgataagaatagcatcttgactctgtccgacaagcggtcaatcatgggcaatcagagccttctttggaagtggaaagggggttctagtttcaccctgcacaagaaactgattgtacctaccgataaagaggccagcaaagcgtggggcagaagtagcacaccggtgttttccttctggctctataacgagaaacctatcgacggttacctgacaatcgattttggggagaagctgatttcaacctccgaagcacaagcgggctttaaagtgaagcttgacttcacaggatggagagccgtaggcgtctctctgaacaatgacctggaaaatagagagatgaccctcaacgctaccaacaccagcagcgacggtacacaggactctattggcaggagtctgggagcaaaagtggatagcatacggttcaaagctcccagtaatgtctctcagggcgagatctatattgaccgcataatgtttagcgtcgatgacgcccgataccagtggtctgactatcaggtgaagacacggctatctgagccagaaatccagtttcacaacgtgaagcctcaactgcccgttacaccagagaacctggccgcgatcgacctcattcggcaaaggctcatcaacgagtttgtgggtggagaaaaagagacgaatcttgccctcgaggagaagatctctaagctgaaatccgactttgatgcacttaacatacacactttagccaatggcggaactcaggggcgacacctcattaccgataagcaaatcatcatctaccaaccagaaaacctcaattcccaggacaaacagctgttcgacaactacgtgattttgggacagtacactacactgatgttccaaatttcccgagcttacgtgctcgagaaagatcctacgcagaaagcccagctgaaacagatgtacctgctgatgacaaaacatctgctcgaccaggggttcgtcaagggctctgctctggttactacacaccactggggatactcatcccgatggtggtacatctccacattgctcatgtcagatgccctgaaggaggcaaatctgcagacccaggtctatgattccctgctgtggtatagtagggagttcaagagttccttcgacatgaaggtatctgcagattccagcgatctggactatttcaatacgttgtccagacagcacctggctctgctgctcctagagccagacgaccaaaagcgcattaacctagtgaacaccttttctcactatatcaccggtgcacttactcaagttcccccaggcggaaaagacgggctcagacccgatggaactgcttggcgccatgagggcaactatcccgggtactcatttcccgctttcaagaatgctgcccagttgatttatttactgcgggataccccgttttcagtgggcgaaagtggatggaataatctgaaaaaagctatggtttccgcttggatttacagcaatccggaagtgggcttacctttggccggccgtcatccctttaacagcccatctctgaaaagcgtggctcagggctactattggctagctatgtcagcgaaatcctctcccgataagactttagcttccatctatcttgccataagcgacaagacccagaatgagagtaccgccatctttggagagactattacgccagcatccctgcctcaaggattctatgcctttaacggtggtgcttttggcatacacaggtggcaggacaagatggtcacactcaaggcctacaatactaatgtgtggtctagcgagatctacaacaaagacaataggtatggtcgctaccagagtcacggagtggcccagatagtcagccaaggttcccagcttagccagggataccaacaggaaggctgggattggaaccggatgcagggggccacgactatacaccttcccttgaaggatctggatagcccaaaaccgcatacactgatgcagaggggcgagaggggtttctccggcacttcaagcttagaagggcagtatggcatgatggcattcgacctgatctatccagcgaatcttgagagatttgaccccaacttcaccgcaaagaagagcgttctcgccgccgacaaccaccttatctttattgggtctaacatcaattctagtgataagaacaaaaatgtggaaactaccttgtttcagcatgccattacgcccaccttgaatacactgtggattaatgggcagaagattgaaaatatgccctaccagacgactctgcaacagggggattggttaatcgacagtaacgggaatggctacctaatcacgcaagccgagaaggtaaacgtcagtcgccaacatcaggtttcagcagaaaacaagaacaggcaacctacagaagggaacttttcatcagcatggatagaccatagcacaagaccgaaagatgccagctacgaatacatggtgttcctggatgctactcccgagaaaatgggagaaatggcccagaagttcagagaaaacaacgggttgtatcaggtgctccgcaaagataaggacgtgcacattatactcgacaagctctcaaacgttacaggatacgccttttaccagccagcttctatcgaggataagtggatcaaaaaggtgaataagccagcaattgtgatgacacatcgccaaaaggataccttgatcgtgtctgcagttacaccagatttgaacatgacccggcagaaagctgctacccctgtcacaatcaacgtcaccattaatgggaagtggcagagcgctgataagaacagcgaagtgaagtatcaggtaagcggggataacactgagctcaccttcacctcctatttcggcatccctcaggagattaagttatcccctctgcct**GGCAGTGGAGAGGGCAGAGGAAGTCTGCTAACATGCGGTGACGTCGAGGAGAATCCTGGCCCA** tctaga‐3′

### 2.4 AAV vector

A shuttle plasmid containing a double‐floxed mCherry under the control of the human synapsin promoter was used for the viral vector backbone, pAAV‐hSyn‐DIO‐mCherry (courtesy of the Bryan Roth laboratory at the University of North Carolina Chapel Hill, commercially available at Addgene, plasmid #50459‐AAV5). The capsid‐encoding plasmid was Rep2/Cap5; pDP5rs (Aldevron Fargo, North Dakota). The ChABC cDNA and P2A sequence were subcloned into the AAV shuttle vector as an Xba I restriction fragment and verified for correct orientation by PCR (Figure 1b). DNA sequencing was carried out to assure fidelity of the shuttle plasmid with the original sequence of the vector and insert. AAV particles were generated by cotransfection of HEK 293 cells with the ChABC shuttle vector, a helper plasmid and capsid encoding plasmid according to a system originally purchased from Agilent Technologies (AAV Helper‐Free System Catalog #240071). Viral particles were harvested from HEK 293 cells, purified and concentrated by ultracentrifugation, and the viral pellet was resuspended in 1× phosphate buffered saline (PBS)/5% glycerol and stored in aliquots at −80°C at a titer of 4.16E+13 GC (genome copies)/ml. The NIEHS Viral Vector Core will provide plasmid constructs for generation of viral particles to qualified investigators upon request with an approved Material Transfer Agreement. The core will also provide viral particles to investigators if the cost of production is provided by the requester.

### 2.5 Virus infusion and tamoxifen treatment

To express ChABC selectively in CA2 pyramidal neurons, first the ChABC‐AAV was injected into the left hippocampus of young adult mice expressing the CreERT2‐fusion protein in CA2 neurons: B6(SJL)‐Tg(Amigo2‐icre/ERT2)1Ehs (*Amigo2*‐*iCre*ERT2+) mice (Alexander et al., 2018). Saline was injected into the right hemisphere for a within‐subject control. To prepare mice for the virus infusion, mice were anesthetized with ketamine (100 mg/kg, intraperitoneal (IP)) and xylazine (7 mg/kg, IP) and placed in a stereotaxic apparatus. An incision was made in the scalp and a hole was drilled over the hippocampal target region for AAV infusion, adjacent to CA2. A 27‐gauge cannula connected to a Hamilton syringe was lowered into the hippocampus (in mm: −2.3 AP, +/−2.5 ML, −1.9 mm DV from Bregma). The undiluted AAV was infused unilaterally at a rate of 0.1 µl/min for a total of 0.5 µl. Saline was infused with the same parameters into the right hemisphere as a control comparison within the same mouse. The cannula was then left in place for an additional 10 min before removing, at which point the scalp was sutured and animals were returned to their cages, singly housed. Mice were administered pre‐operative buprenorphine (0.1 mg/kg, SQ) for pain relief.

After the virus infusion surgery, mice recovered from surgery for 2 weeks. Mice were then administered tamoxifen for 1 day (100 mg/kg dissolved in warm corn oil, IP) to permit Cre activity and induce recombination of the unpacked viral genome. A total of four mice were used for qualitative analysis of ChABC‐AAV expression and PNN degradation. Mice were arbitrarily assigned to two groups (2 per group) that would receive either tamoxifen or vehicle control (corn oil). The number of animals used and the tamoxifen dosing were based on the results of a pilot experiment, in which we found that 3 or 7 days of tamoxifen treatment resulted in a dose‐dependent loss of PNNs. Seven days of tamoxifen treatment is our typical protocol for maximal Cre activation (Alexander et al., 2018). We observed successful targeting of ChABC to CA2 neurons based on mCherry expression and PNN degradation after 3 and 7 days of tamoxifen, however, we observed a widespread loss of PNNs in the ipsilateral hippocampus and in the contralateral hippocampus in the case of 7 days of tamoxifen. We observed minimal effects in the contralateral hippocampus after 3 days, but the loss of WFA stain was still extensive in the ipsilateral hemisphere. This result indicates a dose‐dependent effect of tamoxifen treatment (and thus Cre activation) on ChABC synthesis in this model. We found that 1 day of tamoxifen was effective at Cre activation that resulted in PNN degradation that was better restricted to area CA2. Mice were perfused 2 weeks after the last tamoxifen injection, and tissue was stained for the PNN‐labeling lectin, *Wisteria floribunda* agglutinin (WFA).

### 2.6 Immunofluorescence

For immunostaining, mice were deeply anesthetized with Fatal‐Plus and perfused with cold PBS, followed by 4% paraformaldehyde in PBS, pH 7.4. Brains were removed and post‐fixed overnight at 4°C and submerged in 30% sucrose. Forty‐micrometer‐thick sections were cut on a sliding microtome, blocked in 5% normal goat serum, and incubated overnight at 4°C in biotin‐conjugated WFA (1:1000; Sigma‐Aldrich L1516) to label PNNs, rabbit anti‐PCP4 (1:500, SCBT, sc‐74186) or mouse anti‐regulator of G‐protein signaling 14 (RGS‐14) (1:500, UC Davis/NIH NeuroMab Facility, AB_10698026) to label CA2 pyramidal cells, or guinea pig anti‐ZNT3 antibody (1:500, Synaptic systems #197 004) to label axons from the dentate gyrus (mossy fibers). Sections were washed three times in PBS and incubated in combinations of the following secondary antibodies at 1:500 for 40 min at room temperature: streptavidin Alexa‐488 (Invitrogen #S11226), goat anti‐rabbit A633 (Invitrogen #A21071), goat anti‐mouse A488 (Invitrogen # A11008), or goat anti‐guinea pig A633 (Invitrogen #A21105). Sections were mounted with Vectashield antifade mounting medium with DAPI (Vector Laboratories #H‐1500). Images were acquired on a Zeiss laser scanning confocal (LSM510 NLO) or a Zeiss light microscope using controlled camera settings. Images from the different wavelengths were pseudo‐colored for figure clarity.

## 3 RESULTS

**FIGURE 2:**
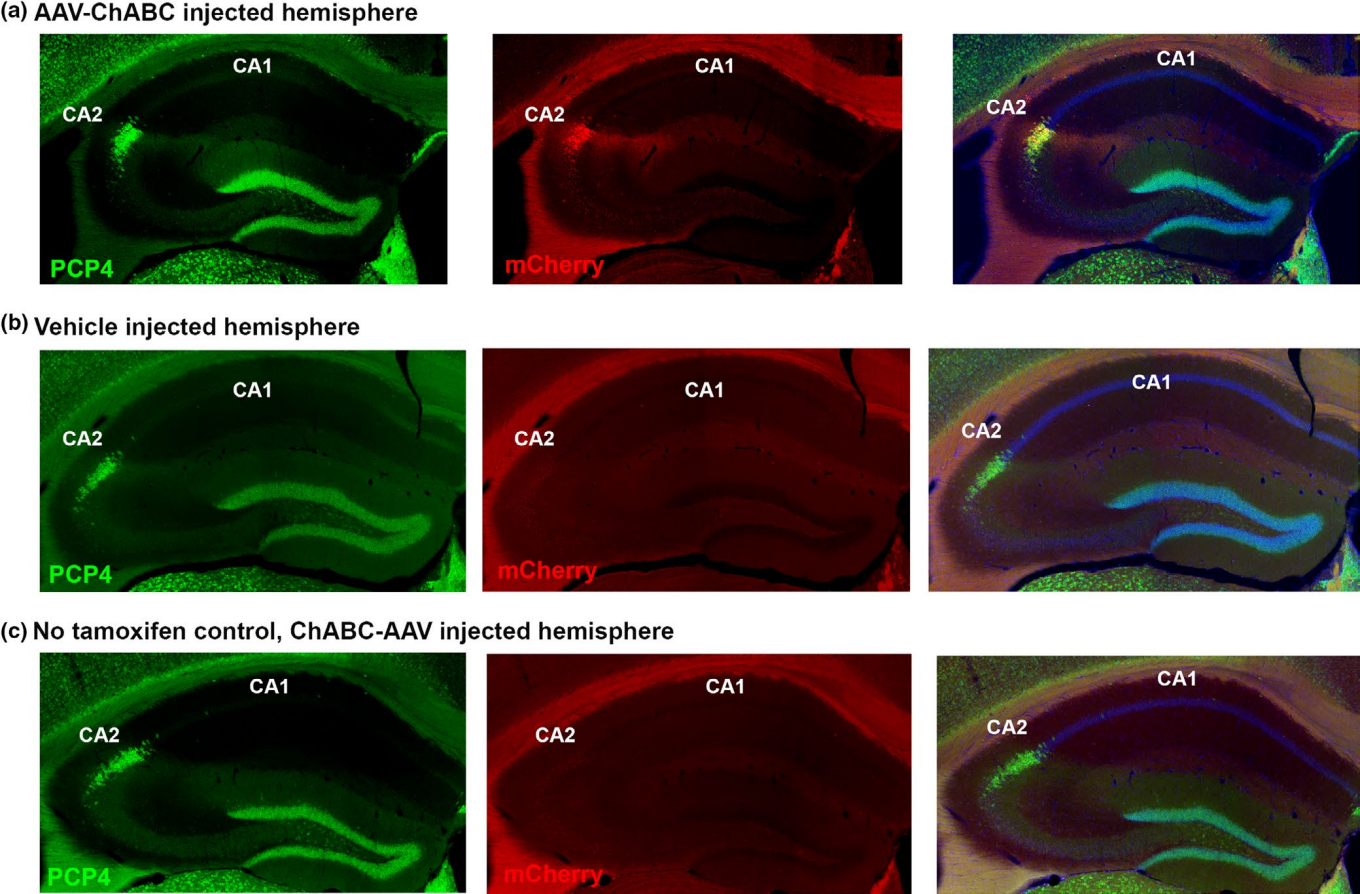
ChABC‐AAV is selectively expressed in CA2 neurons. (a) CA2 neurons, as defined by PCP4 (green, left), are labeled by the mCherry fluorescent reporter (red, center) in the AAV‐ChABC injected hemisphere of a *Amigo2*‐*iCre*ERT2+ mouse. A merged image is shown on the right. (b) The mCherry reporter is not detectable in CA2 neurons of the vehicle injected hemisphere. (c) The mCherry reporter was also undetectable in a no‐tamoxifen control *Amigo2*‐*iCre*ERT2+ mouse, which received AAV‐ChABC and vehicle injections

To target the synthesis of ChABC selectivity to CA2 neurons, we created a Cre‐dependent AAV vector that encodes the PNN‐degrading enzyme ChABC and a cleavable mCherry fluorescent protein. We infused the AAV into the hippocampus of tamoxifen‐inducible CA2 Cre‐expressing mice, *Amigo2*‐*iCre*ERT2+ mice, to selectively target CA2 neurons (Alexander et al., 2018). We infused the contralateral hemisphere with a vehicle (saline) as control. We also added a no‐tamoxifen control *Amigo2*‐*iCre*ERT2+ mouse that received AAV‐ChABC and saline infusions but no tamoxifen treatment. We found that the mCherry fluorescent reporter that is encoded in the AAV construct was expressed unilaterally in the AAV‐ChABC‐infused hemisphere and selectively in CA2 neurons, as defined by the CA2 marker PCP4 (Figure 2a). We did not observe mCherry fluorescence in the vehicle‐treated contralateral hemisphere (Figure 2b), nor did we observe mCherry fluorescence in the no‐tamoxifen control animal (Figure 2c). Thus, we validated that the AAV‐ChABC viral contents were taken up by CA2 neurons and that the mCherry fluorescent reporter was selectively transcribed by CA2 neurons in the hippocampus.

**FIGURE 3:**
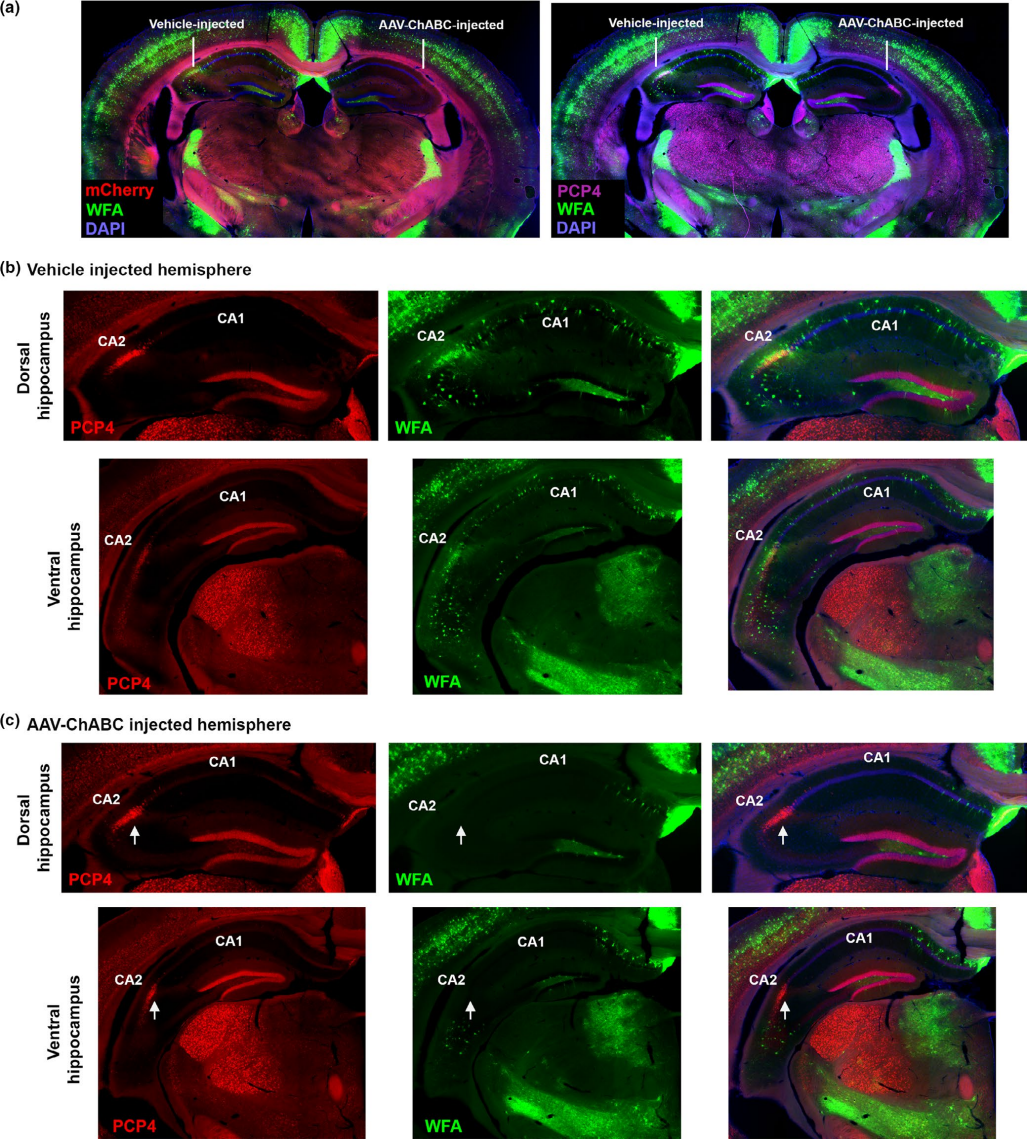
ChABC‐AAV effectively degrades PNNs in area CA2 and projection regions. (a) Whole mouse brain section showing vehicle and AAV‐ChABC injections in each hemisphere with staining for WFA (green), mCherry (red), and DAPI (blue) expression (left), and CA2 localization with PCP4 staining (magenta) in the same section (right). (b) PNNs labeled with the PNN marker, WFA (green, center), are normally expressed in CA2, shown here in the vehicle injected hemisphere, labeled with PCP4 (red, left) in both dorsal (top) and ventral (bottom) hippocampus. Merged images with DAPI are shown on the right. (c) In the AAV‐ChABC injected hemisphere, WFA staining is undectable in area CA2 and in adjacent CA1 and CA3 subregions in dorsal (top) and ventral (bottom) hippocampus, indicating that the CA2 neurons are synthesizing ChABC and ChABC is functionally effective at enzymatic degradation of PNNs in area CA2

Next, we investigated the effects of ChABC on PNN expression in the hippocampus. Two weeks after a 1‐day treatment with tamoxifen, we observed normal expression of PNNs in the dorsal and ventral hippocampus of the vehicle injected hemisphere (Figure 3a,b). In contrast, in the AAV‐ChABC injected hemisphere, we observed a loss of PNNs in area CA2, as well as in areas CA3 and CA1 of both dorsal and ventral regions of the hippocampus (Figure 3b). Because we observed selective localization of the mCherry reporter in CA2 neurons and a loss of WFA staining in CA2 in the AAV‐ChABC injected side, we conclude that this tool is sufficient to degrade PNNs locally in area CA2. We also observed a loss of WFA staining in the neighboring hippocampal subregions, indicating that the enzymatic activity of ChABC extends beyond the CA2 cell bodies, likely due to the ChABC being trafficked and secreted at the projection sites of CA2 neurons in CA1 and CA3. This effect was previously reported in other brain regions using the non‐Cre dependent AAV and lentiviral delivery of ChABC (Alves et al., 2014).

**FIGURE 4:**
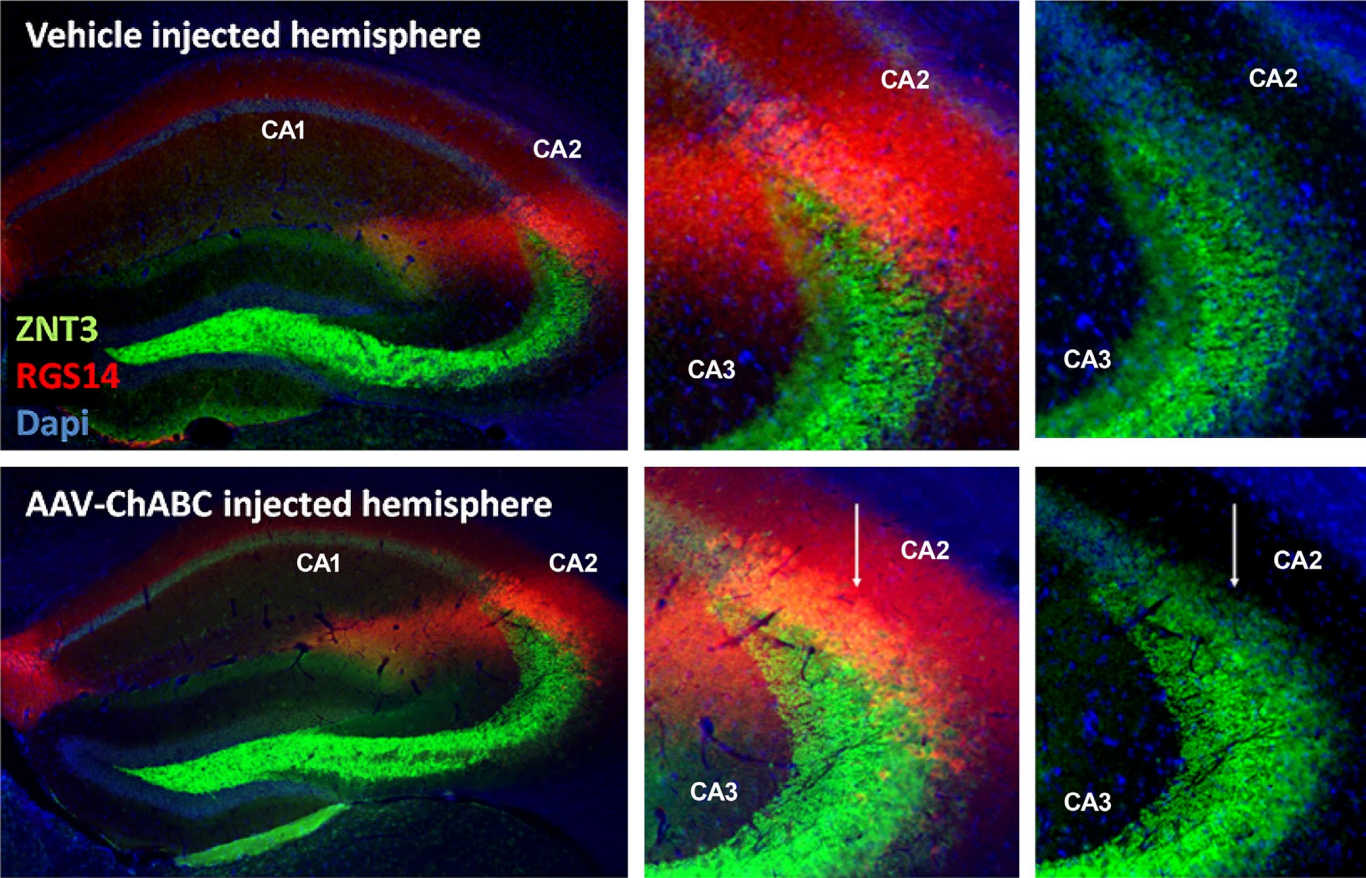
ChABC‐AAV injection in vivo results in mossy fiber sprouting into CA2 stratum pyramidale. Staining for mossy fibers with an anti‐ZNT3 antibody (pseudo‐colored green) labels a larger area 2 weeks after AAV‐ChABC injection compared to vehicle injected hemisphere. Specifically, ZNT3 staining is evident in CA2 SP in the ChABC‐AAV injected hemisphere (top panels, arrows) but faint in the vehicle‐treated CA2 (bottom panels). CA2 cell soma and dendrites are labeled with an anti‐RGS14 antibody (pseudo‐colored red) to indicate CA2/ CA3 border. Cell nuclei are labeled with DAPI (blue). Higher magnifications of the same images are shown on the right, with, and without CA2 (RGS14) labeling shown

Finally, we sought to examine an indicator of structural plasticity that may have resulted from sustained degradation of PNNs. We chose to examine mossy fiber sprouting, which has been reported as an anatomical pathology that is observed in the hippocampus of postmortem patients with epilepsy, where granule cell axons in the dentate gyrus spread into the inner molecular layer of the dentate gyrus (Sutula et al., 1989; Shibley & Smith, 2002). Mossy fiber sprouting has also been linked to ECM components, in that a sustained loss of ECM components has been shown to permit mossy fiber sprouting (Wu et al., 2000). Here we labeled mossy fibers with an anti‐ZNT3 antibody and examined the localization of fluorescence in CA2 and CA1. We observed mossy fiber sprouting in the stratum pyramidale layer of CA2 in the ChABC‐AAV‐injected hemisphere but not in the vehicle‐treated hemisphere, 2 weeks post‐injection (Figure 4). No obvious mossy fiber sprouting was detected in the dentate gyrus of either hemisphere (Figure 4). Taken together, these data present a novel method to degrade PNNs in vivo and suggest that the persistent loss of PNNs in area CA2 permits structural plasticity, including mossy fiber sprouting.

## 4 DISCUSSION

Degrading PNNs with ChABC is a reliable approach for allowing axon regeneration at the site of spinal cord injury (Bradbury et al., 2002). In addition, degradation of PNNs in vivo with ChABC in animal models effectively re‐opens developmental windows of plasticity in several different brain regions. Many experimental limitations in manipulating PNNs in vivo exist, however. First, several groups have worked to create genetic knockouts of components of PNNs. For example, a knockout of the proteoglycan, aggrecan, a major component of PNNs and articular cartilage, is embryonically lethal due to peripheral effects and is limited to in vitro studies (Giamanco et al., 2010). Moreover, loss of a single glycoprotein, such as tenascin‐R, is insufficient to fully reduce staining for this matrix, which is likely due to the redundancy of proteoglycan components (Brakebusch et al., 2002; Carulli et al., 2010; Geissler et al., 2013; Saghatelyan et al., 2001). Recently, a quadruple knockout mouse was generated to address the compensatory effects of ECM components, but expression of PNNs, specifically in area CA2, was still observed (Gottschling et al., 2019). Moreover, the quadruple knockout mouse approach does not allow for cell‐type specificity in targeting PNN loss.

Another approach to degrading PNNs in vivo is by injecting PNN‐degrading enzymes directly into the brain. The most commonly used enzyme for this purpose is the exogenous bacterial enzyme ChABC. Although very effective at degrading PNNs, the enzyme is promiscuous in the mammalian brain and degrades PNNs in a large diffuse area surrounding the site of injection (Pizzorusso et al., 2002; Romberg et al., 2013). Moreover, PNNs are capable of regenerating to baseline levels within 2 weeks after the ChABC injection in the brain (Lensjo et al., 2017). This approach therefore lacks spatial specificity, which limits the study of behavioral effects of PNN function in specific brain regions. Previous studies using viral delivery of ChABC found that indeed cells can synthesize a modified sequence of the bacterial enzyme ChABC (Bradbury et al., 2002; Cafferty et al., 2008; Galtrey et al., 2007), however, these studies still faced limitations by targeting ChABC with cell‐type specificity.

By using a Cre‐dependent construct, we are now able to target the synthesis of ChABC with cell‐type specificity and, in our case, in an inducible manner, degrade PNNs in the targeted region. We successfully expressed ChABC selectively in hippocampal CA2 neurons using our conditional CA2 Cre‐expressing mouse line. The results of our study did, however, reveal limitations in the viral delivery of ChABC. First, degradation of PNNs surrounding the non‐pyramidal neurons in the neighboring CA1 and CA3 regions was observed, suggesting that ChABC‐expressing neurons will still traffic and secrete the enzyme to their terminals. This is very similar to what was previously reported for the non‐Cre dependent ChABC virus, in which viral delivery of ChABC produced widespread effects in both ipsilateral and contralateral hippocampus, in addition to similar outcomes in other targeted brain regions (Alves et al., 2014; Zhao et al., 2011). The authors also noted that the diffusion of the AAV vector is poorly constrained (relative to control AAV‐GFP injections), suggesting that the ChABC effects are likely a combination of AAV vector diffusion *and* ChABC secretion from axonal projections. Accordingly, the degradation effects of our Cre‐dependent AAV‐ChABC is likely a result of local/ axonal diffusion of ChABC and *not* a result of vector diffusion, as validated by our localized mCherry expression. Second, we noted a dose‐dependent effect of tamoxifen on the synthesis of ChABC. We observed a dose of once per day for 3 or 7 days of tamoxifen resulted in a substantial spreading of PNN degradation where PNNs were degraded in the vehicle‐injected contralateral hippocampus (data not shown). Overall, this approach is advantageous in (a) targeting ChABC synthesis to a specific cell‐type and (b) addressing limitations of PNN regeneration such that ChABC synthesis and PNN degradation is sustained over time after Cre recombination. Note that the small number of animals in each case (two in each group) precluded quantitative analysis. Nevertheless, these data, combined with our pilot studies using higher tamoxifen doses and robustness of the evident effect, showed that qualitatively, PNNs were lost and staining for mossy fibers was increased in the stratum pyramidale. Lowering the dose of tamoxifen even further, or lowering the viral dose, in the case of studies using non‐inducible Cre, could further restrict the effects or spread of the ChABC, but effects would depend on the specific cell types studied. Future studies will be required to determine the effects of this construct on the integrity of specific PNN components and how they may be altered over time.

In conclusion, this novel method is effective at targeting neurons in vivo with cell‐type selectively and overcomes limitations related to PNN regeneration over time. Moreover, the observed mossy fiber sprouting is also indicative of functional outcomes, a finding that is consistent with a previous study that observed regeneration of lesioned corticospinal axons after treatment with the non Cre‐dependent ChABC virus (Zhao et al., 2011). PNNs have been heavily implicated in several neurological diseases such as schizophrenia, bipolar, prion disease, epilepsy and Creutzfeldt‐Jakob disease (Belichenko et al., 1997; Leontovich et al., 1999; Moleres & Velayos, 2005). The pathology of PNNs is complicated, however. For example, PNNs are reportedly up‐ or down‐regulated after seizure in the hippocampus (McRae et al., 2010, 2012), and degradation of PNNs results in an increase in seizure susceptibility (Rankin‐Gee et al., 2015). In fact, ChABC treatment can have profound effects on CA2 synaptic plasticity, excitability, and interneuron connectivity (Carstens, et al., 2016; Hayani, et al., 2018). Thus it remains unclear whether alterations in PNNs precedes or follows the onset of disease. The inducible viral method described here may help address this question. This novel approach will allow the targeting of ChABC synthesis with cell‐type selectivity in vivo and may ultimately reveal how long‐term loss of PNNs may disrupt cell health and/or cause behavioral deficits such as learning and memory impairments (Babb et al., 1991; Belichenko et al., 1997; Berretta, 2012).

## CONFLICT OF INTEREST

The authors declare no conflicts of interest.

## AUTHORS’ CONTRIBUTIONS

KEC, BG, and GMA performed experiments. KEC analyzed the data. KEC, BG, GMA, and SMD designed and coordinated the investigations. KEC wrote the manuscript with input from BG, GMA, and SMD.

### Peer Review

The peer review history for this article is available at https://publons.com/publon/10.1111/ejn.15050.

## Data Availability

Original images are available from the corresponding author, [S.M.D], upon reasonable request.
